# pH-triggered conduction of amine-functionalized single ZnO wire integrated on a customized nanogap electronic platform

**DOI:** 10.1186/1556-276X-9-53

**Published:** 2014-01-31

**Authors:** Valentina Cauda, Paolo Motto, Denis Perrone, Gianluca Piccinini, Danilo Demarchi

**Affiliations:** 1 Center for Space Human Robotics, Istituto Italiano di Tecnologia, Turin 10129, Italy; 2 Department of Electronics, Politecnico di Torino, Turin 10129, Italy

**Keywords:** ZnO wire, Aminopropyl functionalization, Nanogap electrodes, pH-responsive behavior, Gold-ZnO-gold junction

## Abstract

The electrical conductance response of single ZnO microwire functionalized with amine-groups was tested upon an acid pH variation of a solution environment after integration on a customized gold electrode array chip. ZnO microwires were easily synthesized by hydrothermal route and chemically functionalized with aminopropyl groups. Single wires were deposited from the solution and then oriented through dielectrophoresis across eight nanogap gold electrodes on a platform single chip. Therefore, eight functionalized ZnO microwire-gold junctions were formed at the same time, and being integrated on an *ad hoc* electronic platform, they were ready for testing without any further treatment. Experimental and simulation studies confirmed the high pH-responsive behavior of the amine-modified ZnO-gold junctions, obtaining in a simple and reproducible way a ready-to-use device for pH detection in the acidic range. We also compared this performance to bare ZnO wires on the same electronic platform, showing the superiority in pH response of the amine-functionalized material.

## Background

Over the last decade, zinc oxide (ZnO) was intensively studied due to its promising catalytic, electrical, wetting, and optical properties [1-3], shading light on several technological applications, including photovoltaic cells [4], nanogenerators [5,6], field-effect transistors [7], gas [8] and strain sensors [9], and other electronic nanodevices [10]. It is a unique material exhibiting wide bandgap (3.37 eV) [11], large exciton binding energy (60 meV) [12], and low lasing threshold, applicable to optoelectronics, sensors, transducers, and nanogenerators [13-16]. Several efforts were therefore focused on the preparation and characterization of ZnO materials at the sub-micrometric scale and with different morphologies, including micro- and nanowires, multipods, and nanoparticles [2]. One-dimensional structures can be easily connected to electrodes for exploiting the semiconducting properties and enabling their study as chemical or biological sensors [17,18]. In particular, ZnO wires were used for constructing pH-sensing devices, since the surface electrical charge density of ZnO changes with pH in electrolyte solutions. In general, the pH response of metal oxide surfaces is attributed to the formation of hydroxyl groups, changing the net surface charge as a function of the pH value [19]. This results in a voltage variation at the interface between the semiconductor and the liquid [20,21]. Based on this principle, ZnO nanorods were used to fabricate a highly sensitive pH sensor on Femtotio® II capillaries to detect the intracellular pH of a human fat cell [22]. Other authors [23] showed pH-sensing devices based on single ZnO nanorods with Ohmic contacts at either ends, exhibiting slight changes in current (about 5 nA at 0.5 V per pH unit) upon exposing the surface to liquid electrolytes. The device sensitivity was also enhanced by exposing ZnO to UV light, thus increasing the measured conductance at a certain pH with respect to the same experiment under dark conditions.

**Figure 1:**
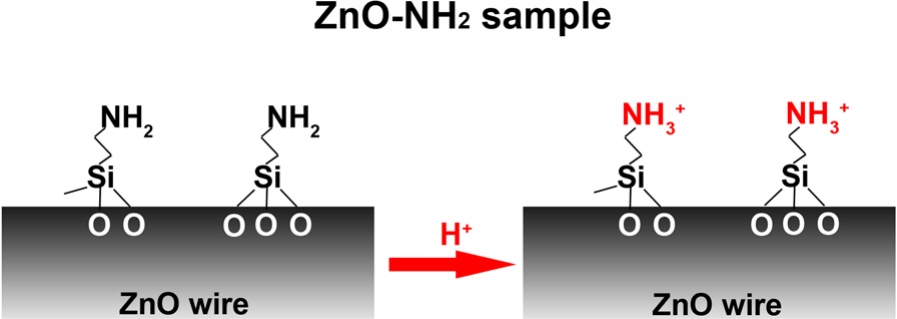
**The chemical structure of the amine shell on the ZnO wires.** The pH-responsive structure is attributed to the reversible protonation mechanism of the amine groups.

Here, we report on a large increase of the current in the order of microampere at 2 V (or of one order of magnitude of the conductance at 0.75 V) measured from a single ZnO microwire, in response to a reduction from neutral to acid pH. This enhanced response was significantly higher than those reported in the previous literature and was obtained thanks to the functionalization of the ZnO wires with a shell of aminopropyl groups (ZnO-NH_2_), which are highly responsive to pH variation due to protonation/deprotonation mechanism of the ending -NH_2_ group (Figure 1). The functional wires were aligned by dielectrophoresis among eight nanogap gold electrode array chips. This resulted in eight parallel gold-ZnO-gold junctions at the same time on a single chip integrated on a ready-to-use electronic platform. We measured a remarkable change of the current as a function of the solution pH and the acid concentration in contact with the chip, as a result of the ion-induced changes of the surface potential of our ZnO-functionalized wires. The simulations of the experiment confirmed our results. We also compared this behavior to the non-functionalized ZnO wires deposited on the same electronic platform and to the literature results on ZnO [23], thus showing the superiority in pH response of our amine-functionalized material. The amine groups are often used as further anchoring moieties for molecules or metals having biological [24-26], catalytic [27,28], imaging [29], or optical purposes [30]. Therefore, these results suggest that amine-functionalized ZnO structures deposited on an electrode array chip can be a very promising platform for a wide variety of sensing applications. The innovation of the presented approach lies in the integration of the single amine-functionalized wires on a nanogap electrode chip and the parallel current–voltage characterization and pH sensing measurements of the eight ZnO-gold junctions. This can be the first step toward a smart and portable micro-chip sensor [31]. The ease and reproducible synthesis of the functionalized ZnO wires as well as on the cost-effective and simple fabrication of the nanogap electrode chip are also further advantages of the presented system. In addition, the customized electronic board developed in this work allows several *in situ* operations: (1) the nanogap fabrication from photolithographed gold probes, (2) the ZnO single wire alignment among the nanogap though dielectrophoresis, and (3) the ZnO-metal junction electrical testing as such and under pH variation. The main goal of this work is therefore to prepare and test a nanoscale device, correlating the strong relationship between the surface chemistry of the functionalized ZnO material and the ZnO-gold electrical conductance.

## Methods

### Synthetic procedures

The ZnO microwires were synthesized, modifying a previous synthesis [30], by slowly dropping 1.48 g zinc nitrate hexahydrate Zn(NO_3_)_2_?·?6H_2_O (5 mmol, Sigma-Aldrich S.r.l. Milan, Italy) in 10 mL bidistilled water (Direct Q, Millipore Co., Billerica, MA, USA) into 3.35 g potassium hydroxide (60 mmol, Merck KGaA, Darmstadt, Germany) in 10 mL water under vigorous stirring. The transparent solution was then transferred in a closed Teflon vessel and placed in an oven at 70°C for 5 h. Afterwards, the formed ZnO microwires were collected by filtration, washed thoroughly with water until neutral pH was reached, and dried in air at 60°C.

Post-grafting with aminopropyl groups on the ZnO microwires was carried out with 10 mol% of the functional moiety with respect to ZnO molar amount. In detail, 250 mg (3.075 mmol) of ZnO microwire was outgassed for 2 h in a round flask connected to a Schlenk line. Then, the atmosphere was changed to nitrogen, 10 mL of dry toluene and 0.307 mmol of aminopropyltrimethoxysilane (APTMS; 55.04 mg) were added, and the solution was refluxed for 24 h under nitrogen. The functionalized microwires (ZnO-NH_2_) were washed with acetone and isopropanol and then dried at 60°C overnight (Figure 1, left).

### Characterization

Morphological and structural characterizations were carried out by field emission scanning electron microscopy (FESEM; Dual Beam Auriga from Carl Zeiss AG, Oberkochen, Germany) and by X-ray diffraction patterns with an X’Pert diffractogram (CuK_α_?=?1.54 Å) in Bragg-Brentano configuration. Fourier transmission infrared (FTIR) spectroscopy was carried out in attenuated total reflectance (ATR) on a Bruker Equinox 55 (spectra are baseline substracted; Bruker Optics Inc., MA, USA). Nitrogen sorption measurements were obtained at 77 K from Quadrasorb instrument (Quantachrome Instruments, Boynton Beach, FL, USA). The Brunauer-Emmett-Teller (BET) surface area was measured by multipoint method within the relative pressure range of 0.1 to 0.3 p/p_0_. Thermogravimetric analysis was performed on a Netzsch STA 440 Jupiter thermobalance (heating rate of 10 K min^-1^ in a stream of synthetic air of about 20 mL min^-1^; Verona, Italy).

### Nanogap array chip fabrication and setup

**Figure 2:**
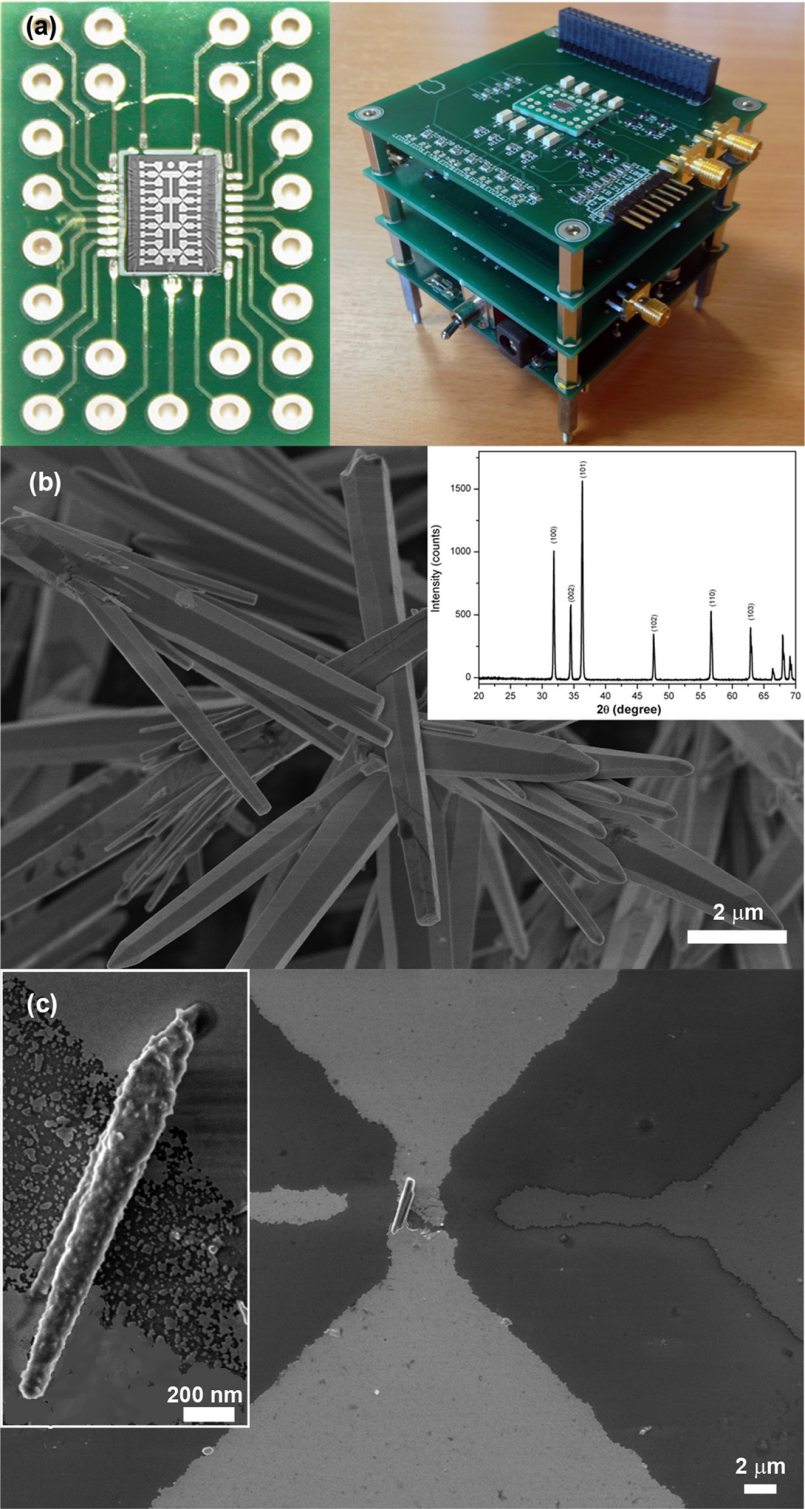
**The nanogap array platform and the FESEM image of the ZnO microwires. (a)** The gold electrode array chip, having eight nanogaps, mounted on the PCB (left) and the customized nanocube electronic board (right). **(b)** FESEM image of the ZnO microwires with X-ray diffraction pattern. **(c)** Amine-functionalized ZnO-NH_2_ wires dielectrophoretically aligned across the nanogap, bridging the two gold electrodes.

The nanogap array platform for ZnO wire positioning and testing was prepared by conventional photolithography, obtaining eight gold wires (25-nm thin, 6-mm long, and 2-mm wide), distributed in two columns with four parallel wires each, on Si wafer covered with 200 nm of silicon dioxide (Figure 2a, left) [32]. The rupture of the gold wire was obtained by the electromigration-induced break junction (EIBJ) method [33,34]. The whole nanogap array platform consisted of a central silicon chip (2.4?×?4.1 mm), bonded to a customized printed circuit board (PCB, 10?×?20 mm). The bonding wires were incorporated in a polydimethylsiloxane ring, which was used for protecting and insulating the bonding wires and confining the ZnO wire suspension during the deposition.

Both the ZnO and ZnO-NH_2_ microwires were suspended in isopropanol (0.2 mg/mL) and after a 10-min sonication, one drop of the suspension was dispensed on the eight-nanogap array chip. Dielectrophoresis (DEP) of the microwires was carried out at 20-MHz AC signal and 3 *V*_pk-pk_ (sinusoidal waveform, offset 0 V) until the complete evaporation of the solvent took place.

Simulation of the *I*-*V* characteristics was carried out using the non-equilibrium Green’s functions (NEGF; Atomistix ToolKit (ATK), QuantumWise A/S, Copenhagen, Denmark) [35-37], based on the DFT model, to obtain a full *ab initio* self-consistent description of the transport properties of the ZnO-gold junction under finite bias conditions.

## Results and discussion

### Material characterization

The reproducible and scalable hydrothermal synthesis produced ZnO microwires with typical length of 2 to 10 μm and a diameter of 200 to 600 nm (as observed by FESEM in Figure 2b). The X-ray diffraction pattern (inset of Figure 2b) shows the reflection typical of a wurtzite crystalline structure of the microwires (JCPDS 80–0074, *a*?=?0.3253 nm, *c*?=?0.5215 nm, hexagonal symmetry, space group *P*63*mc*). In addition, the sharp diffraction peaks indicate that the product has a high purity and high degree of crystallinity.

**Figure 3:**
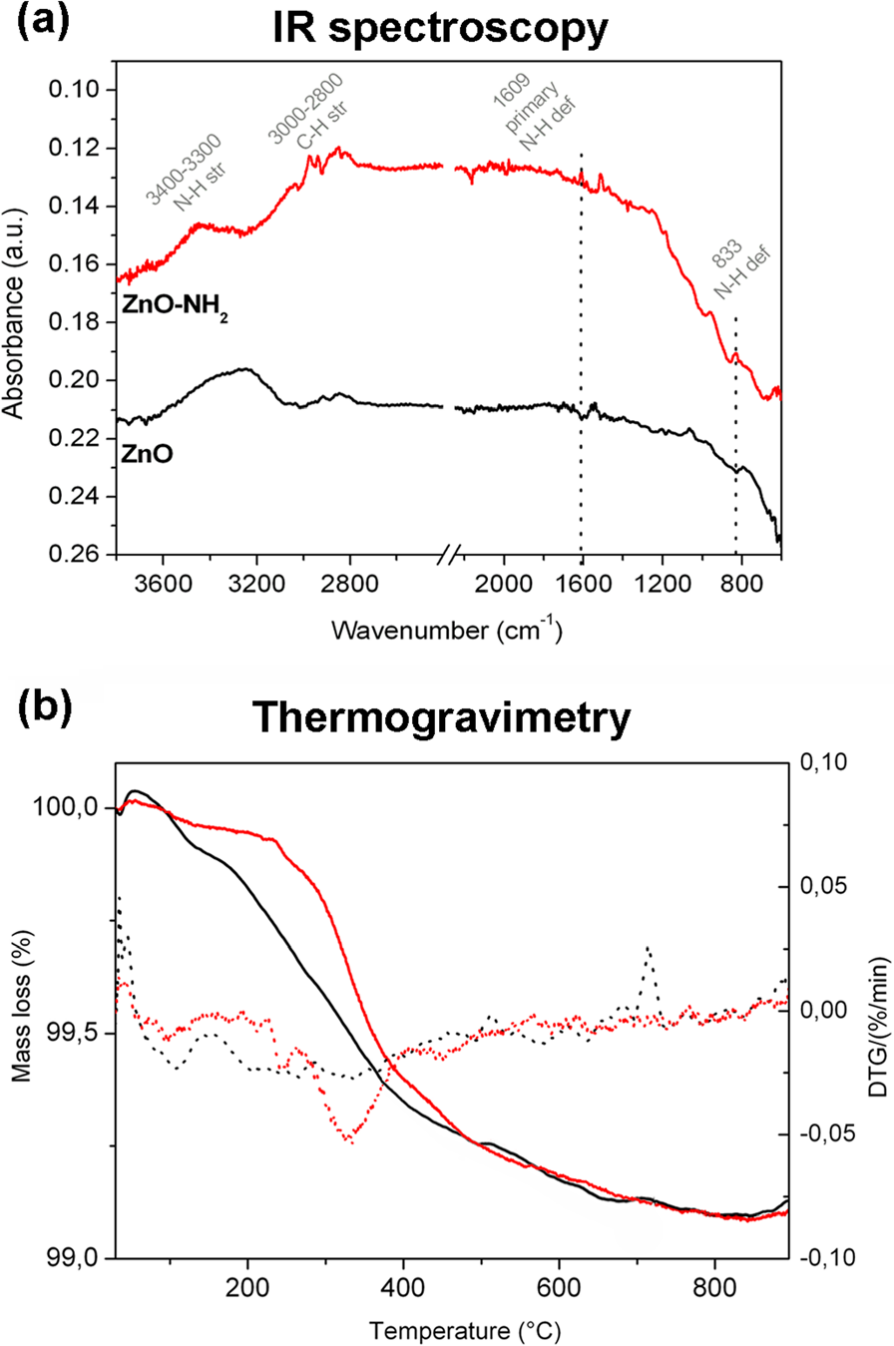
**Fourier transform infrared spectroscopy and thermogravimetric analysis of ZnO. (a)** Fourier transform infrared spectroscopy and **(b)** thermogravimetric analysis on the ZnO (black lines) and amino-functionalized ZnO (red lines) samples.

The surface of the ZnO wire after the chemical functionalization became covered by an organic layer, i.e., the amine groups (Figure 2c), whereas it was clean prior to the chemical treatment (Figure 2b). Additional evidence of aminopropyl groups resulted from both thermogravimetric and infrared spectroscopy measurements. Figure 3a shows the FTIR spectra of both ZnO (in black) and ZnO-NH_2_ (in red) for easy comparison. The bare ZnO wires show the presence of hydroxyl groups (at 964 and 796 cm^-1^, asymmetric bending and stretching vibration of Zn-OH). Furthermore, a broad band at 3,600 to 3,100 cm^-1^ corresponding to water and hydroxyl groups on the wire surface can be observed. The peak at 1,629 cm^-1^ indicates the bending modes of the water molecules adsorbed on the surface of the ZnO material. In the ZnO-NH_2_ spectrum, the deformations of primary amine (N-H) are located at 833 and 1,609 cm^-1^. The band between 3,500 and 3,300 cm^-1^ corresponds to the N-H stretching vibration, from 3,000 to 2,800 cm^-1^ to the stretching vibration of the C-H groups, belonging to the propyl chain.

A tentative quantification of the aminopropyl groups is based on thermogravimetry (Figure 3b) and the available surface area (0.96 m^2^/g) of the ZnO wires, as calculated by the BET model from nitrogen sorption measurements (as reported in Additional file 1: Figure S1). The weight loss of the functionalized sample is slightly higher with respect to the sample with unfunctionalized ZnO, in particular, the first derivative of the thermogravimetric curve (DTG, red dot curve) shows a peak from 300°C to 400°C, indicative of the loss of organic materials. The weight loss in this temperature range can be generally attributed to the materials adsorbed or anchored to the ZnO surface, including the amine functionalizing agent. Calculation based on the weight loss of both samples returns a value of about 2 μmol/g of sample (0.37 mg/g) of organic material; thus, in absence of any contamination, one could assume this value as the maximum amount of aminopropyl group attached to the surface. By taking into account the value of specific surface area, the hypothetic maximum aminopropyl group density is about 0.38 mg/m^2^ or 1.78 molecules/nm^2^. From the thermogravimetric curve, we also calculated about 2.11 mg/g (2.19 mg/m^2^) of hydroxyl groups on the bare ZnO surface (black curve), whereas after the functionalization with APTMS, the groups are reduced to 1.17 mg/g (1.22 mg/m^2^). This decrease of hydroxyl group is clearly attributed to the effective anchoring of the aminopropyl groups to the ZnO surface, since an average of two/three methoxysilane ending groups of the APTMS molecule condense with the respective hydroxyl group on the ZnO surface during the functionalization reaction (Figure 1, left).

All these findings, combined with the FTIR spectroscopy, confirm the successful functionalization of ZnO with aminopropyl groups. In addition, the reduction of the hydrophilic hydroxyl groups on the wire surface after functionalization leads a useful indication about the degree of wettability of the ZnO and ZnO-NH_2_ surfaces. An easy experiment, confirming the surface charge of the two different samples, relies on the pH-triggered transfer of the ZnO wires from hydrophobic to hydrophilic solutions in a biphasic system (organic solvent and water) and is reported in Additional file 1: Figure S2.

### Nanogap array platform setup

The nanogap array platform for ZnO wire positioning and testing was prepared by conventional photolithography. To have a useful platform where to produce the nanogaps, a silicon chip (2.4?×?4.1 mm in size) containing eight gold butterfly probes was obtained by photolithography as shown in Figure 2a (left) [32]. The chip was also wire-bonded to a PCB. In this way, eight nanogap structures can be obtained on the same chip by EIBJ method [33,34] with a gap final size ranging from 10 to 200 nm. Because of the system configuration, each nanogap electrode on the chip is independent; therefore, a high number of measurements is individually achievable.

The nanogap array platform was designed to easily interface the ZnO-gold junctions with the external instruments and electronic apparatus in a plug-and-play method, being ready for *in situ* measurements. The nanogap chip on the PCB was indeed integrated on a modular, flexible, and low-cost electronic system (nanocube, Figure 2a, right), which implements the hardware-software (HW-SW) apparatus for both the complete fabrication and characterization of the nanogap, based on an *ad hoc* and efficient EIBJ algorithm. This modular approach is quite innovative and permits a continuous updating and improvement of the sub-systems, each dedicated to different tasks. In particular, the nanocube system consists of the following:

1. A driver module which drives the gold probes and provides enough input voltage swing for the nanogap EIBJ fabrication process. During the deposition and the characterization of the ZnO microwires, it provides both DC and AC voltage signals.

2. A measure module, performing real-time measurements of the current flowing into the gold probe (hence to evaluate resistance variations), from hundreds of milliampere (when the current is high and the gap is not yet created) to some nanoampere (immediately after breaking the sample, e.g., tunneling current). This range is also suitable to perform the current measurements for ZnO-gold junction characterization.

3. A switch module through which the PCB cartridge is connected to the nanocube system. To enable probe multiplexing, it includes eight optically isolated relays so that we can individually select each gold probe. This permits to electromigrate and characterize the probes one by one, thus allowing to run the measurements on all nanogaps individually without altering the setup.

4. A control module that is a Linux (San Francisco, CA, USA) embedded processor-based board controlling all the system features. This micro-programmed unit has sufficient performance and provides a large number of communication interfaces which can control the modules described above. The Linux architecture was designed using an open embedded build framework (OE); however, it can run a custom tweaked and patched deterministic and real-time kernel. This option can control both the fabrication and characterization processes with real-time measurements. This module implements also the electromigration algorithm. Finally, all the experimental data are collected by this module and transmitted to a host device (e.g., a computer or a tablet) through a wireless IEEE 802.11 WLAN link. This feature allows placing the system in a controlled environment (clean room) and allows the user to operate in a separate area.

The described system is indeed designed and conceived to enable ease of operation in both electronics and materials science laboratories, thanks to a customized assembly of PCB cartridges, designed to achieve a complete control of the gold probes to be electromigrated [33,38]. Moreover the whole nanogap array platform was fabricated with low-cost components [33] and can be easily disconnected and washed several times to remove the ZnO wires. It is possible to perform wet analysis too, by just spin coating or drop casting the solution that has to be measured on the chip and then connecting it to the nanocube board. The butterfly nanogap array is also arranged in a way to allow the chip integration with microfluidic channels (here not exploited). The nanogap array platform is therefore reusable for different purposes and easily portable, thus giving the possibility to be characterized directly with several instruments, i.e., cryostats for very low temperature measurements, or Raman microspectroscopes for *in situ* characterization [38] or AFM, STM, and FESEM microscopes (as in Figure 2c) for direct measurements, also under vacuum conditions.

In order to deposit the wires across the nanogaps, DEP [39,40] was carried out, leading to the prompt alignment of single microstructures across the desired gold electrodes, thus bridging the nanogaps (Figure 2c). This deposition process led, at the same time, to eight gold-ZnO-gold junctions on a single chip. Further washing steps in water or organic solvents (i.e., isopropanol) did not remove the deposited ZnO wires, unless sonication was applied for at least 10 min. It was indeed reported [41] that DEP can induce a local melting of the gold electrode, thus strongly binding and electrically connecting the ZnO wire.

### Electrical characterization

**Figure 4:**
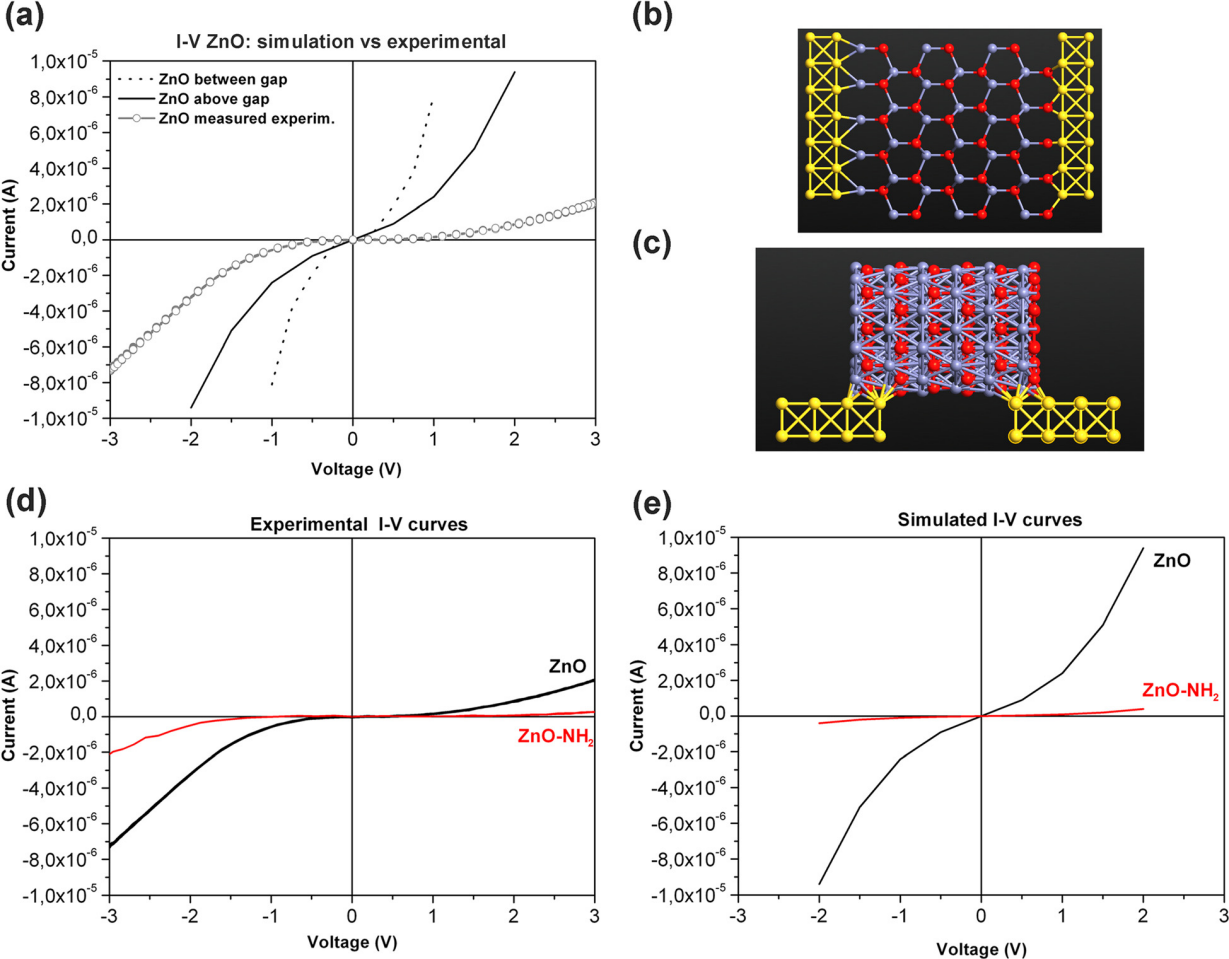
**Experimental and simulated*I*-*V*curves. (a)***I*-*V* characteristics for the ZnO wire-gold junctions obtained experimentally (empty circles), in comparison with the simulated curves, where ZnO is either placed on the gold electrodes (straight line) or between them (dot line). Atomistix toolkit (ATK) scheme of ZnO between the gold electrodes (**b**, top view) or on them (**c**, lateral view). **(d)** Experimental and **(e)** simulated *I*-*V* of the ZnO-gold junction (black line) and of ZnO-NH_2_-gold one (red line).

Prior to the pH measurements, both the ZnO and ZnO-NH_2_ single wires on the nanogap platform were measured in DC in dark at room temperature (Figure 4d). Non-linear *I*-*V* characteristics, showing an asymmetric rectification typical of Schottky contact between ZnO and gold, were obtained for both sample types. The rectifying behavior is attributed either to the metal junction or to the alternating zinc and oxygen planes along the *c*-axis, leading to a dipole moment and thus to the asymmetry of current flow along the wire axis [41]. The *I*-*V* curves obtained for each single ZnO-gold junctions on the same chip are all similar to each other; for simplicity, here, we show only an exemplar one.

The current from the ZnO-NH_2_-gold junctions is remarkably lower than that of the unfunctionalized ZnO-gold ones). The flattening of the *I*-*V* curve is attributed to the high resistive behavior of the propyl chain (as depicted in Figure 1) grafted to the zinc oxide surface.

The ATK simulation of the *I*-*V* characteristics was carried out by positioning the bare ZnO structure both between the gold electrodes (Figure 4b) and on them (Figure 4c). The transport properties are determined by the electronic structures of the wires and electrodes. We assumed a two-probe device with ZnO wire connected to two semi-infinite Au(001) electrodes. The initial hexagonal cross-section of ZnO was cut from a large wurtzite supercell along the [0001] *c*-direction. The two-probe device was an open system, consisting of three parts: the two electrodes and the ZnO scattering region. The left and right regions consisted of four layers of Au(001)-6?×?6 surface atoms, repeated periodically, forming the infinite electrode. The scattering region included a portion of the semi-infinite electrodes where all the screening effects take place. Therefore, the charge distribution of the electrodes corresponded to the bulk gold phase with a prescribed numerical accuracy. Figure 4b shows a three-cell wire sandwiched between the electrodes, where each unit cell of ZnO consists of 20 O^–^ and 20 Zn atoms (more details in the Additional file). This method was similar to those used in the literature for carbon and boron nitride nanotubes, and OPVn molecules [42-44], maintaining fixed distances to compare the transport properties of 1D nanostructures with different lengths.

The simulated *I*-*V* plot shows a semiconducting-like behavior (Figure 4a, dot line), confirming both the experimental results and those reported in the literature [45]. With the same bulk configuration, we performed a second simulation with the wire placed on the gold electrodes (Figure 4a, solid line, and scheme in Figure 4c), also reflecting the Schottky-type electronic structure discussed above. This second configuration shows a current decrease for the same applied voltage with respect to the first case (wire between). This occurred because the interface was reduced and deflected about 20%. Both simulated *I*-*V* curves show a higher current at the same voltage with respect to the experimental *I*-*V*. This is attributed to the non-perfect contact of the wire with the gold electrodes in the real case, also observed by the asymmetry of the experimental plots.

### pH-triggered conduction of the ZnO-metal junctions

**Figure 5:**
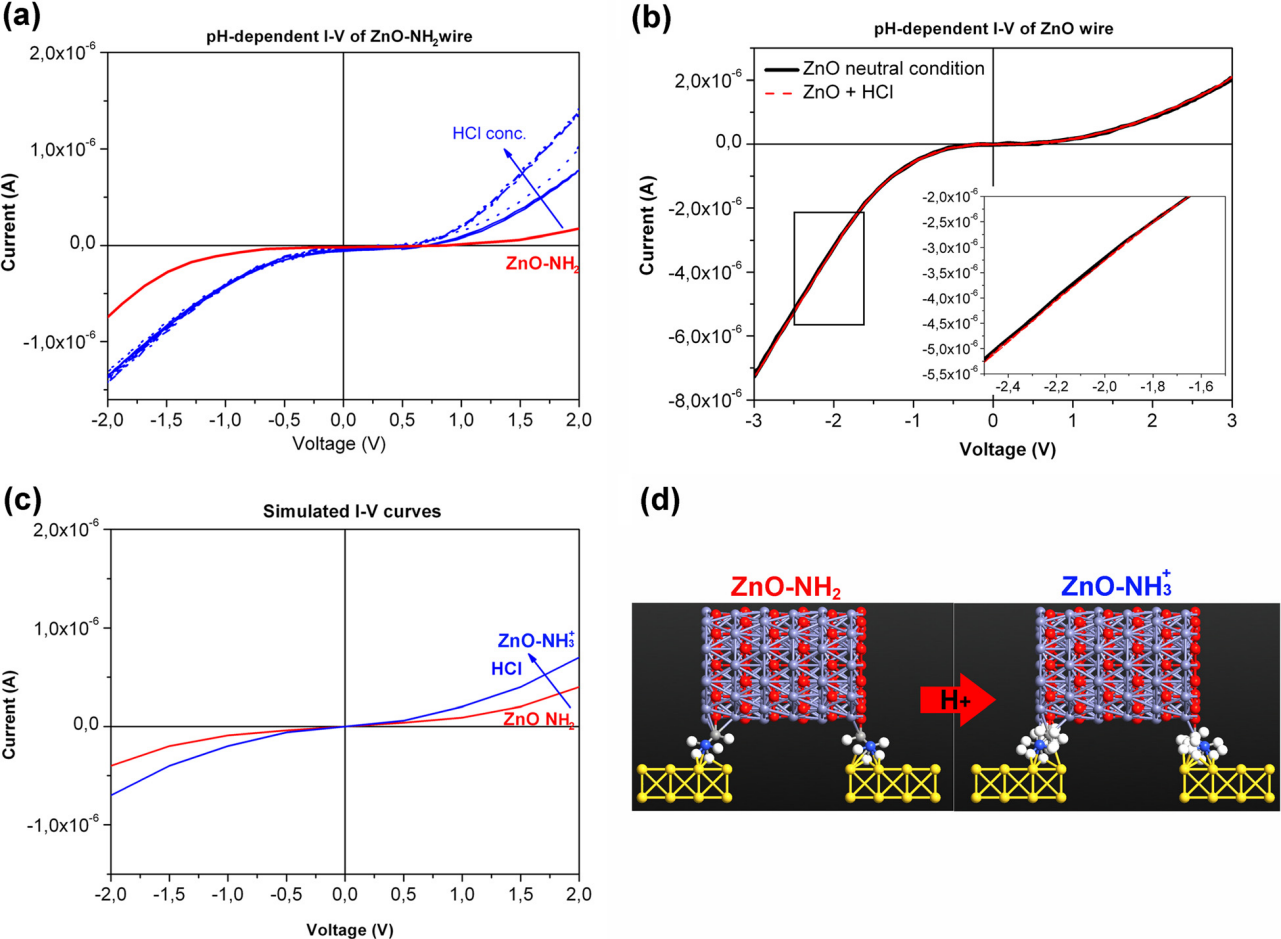
**pH-triggered*I*-*V*curves for the amine-functionalized ZnO-gold junctions. (a)** Experimentally recorded in neutral conditions (red line) and after addition of HCl at pH 5 (straight blue line), pH 4 (dot blue line), and pH 3 (dash-dot blue line). **(b)***I*-*V* in neutral (black) and acidic (red) conditions of the unfunctionalized ZnO-gold junction. **(c)** Simulated *I*-*V*. **(d)** ATK schemes of the ZnO-NH_2_ material on the gold electrodes before (ZnO-NH_2_) and after acid protonation (ZnO-NH_3_^+^).

pH-dependent conduction measurements were carried out on both amine-functionalized and unfunctionalized ZnO wires by injecting a drop (10 μL) of mild acid (10 μM HCl, pH 5), letting the solution act for few seconds, and drying it under nitrogen flux. It has to be noted that all the acid concentrations used are not enough to dissolve the ZnO structures, and no evidence of degradation due to any chemical reaction after contact with acidic pH solution was experienced. Interestingly, the reduction of the pH from 7 to 5 on the ZnO-NH_2_ wire triggers a shift towards higher absolute values of the measured current of the whole *I*-*V* characteristic (straight blue line, Figure 5a), with respect to the *I*-*V* in neutral conditions (red curve). At 2 V, the shift was of 0.52 μA. A further pH reduction using a higher HCl concentration (100 μM, pH 4, dot blue line and further 1 mM, pH 3, dash-dot blue line) brings to even higher positive values of the current (relative shift of 0.84 and 1.15 μA at 2 V, respectively). This pH sensing resulted from the dramatic change in the charge state of the amine, as it gained protons in response to the pH of the surrounding medium. The isoelectric point (IEP) of the aminopropyltrimethoxysilane grafted on an oxide surface is at a pH slightly above 5, as previously verified [25,46]. Therefore, at the pH values experienced in this work, the amine groups are positively charged (shifting from -NH_2_ to -NH_3_^+^, see Figure 1). After abundant washing with water, the *I*-*V* restored back to the initial neutral conditions. The results were observed on different amine ZnO-gold junctions throughout the whole chip or on different gold electrode chips, showing the repeatability of the system. Additionally, the pH-triggered conduction variation can be obtained for several cycles up to ten times, without any damage of the ZnO structure.

To confirm theoretically this behavior, we run a second simulation, using the configuration with ZnO on gold electrodes, by inserting the amino groups between the gold electrodes and the ZnO wire (see the ATK scheme in Figure 5d, left). The new simulated *I*-*V* (red lines in Figure 4) showed a sharp decrease of the absorbed current with respect to that of bare ZnO (Figure 4e), as also observed for the experimental curves (Figure 4d). Due to the presence of the amino groups at the interface, the energy required to promote electrons in the conduction band is higher than that in the unfunctionalized configuration; thus; the inflection point of the ZnO-NH_2_*I*-*V* curve is shifted along the *x*-axis with respect to that of the ZnO *I*-*V*. It is thus necessary to provide a higher voltage to activate the exponential increase of the absorbed current.

To simulate the action of the acid on the amine-functionalized ZnO, H^+^ ions were added to the amino groups with the ATK software package (Figure 5d, right). The simulated *I*-*V* (Figure 5c, blue curve) showed an increase of the current at the same bias voltage, as also reported experimentally in Figure 5a. Therefore, the addition of acid causes the increase of absorbed current in a consistent manner to the experimental phenomenon, confirming the system capability toward pH sensing. Compared with the experimental curves, the simulated absorbed current is slightly lower, since the simulated surface of the amino groups is much smaller than that of the real one.

**Figure 6:**
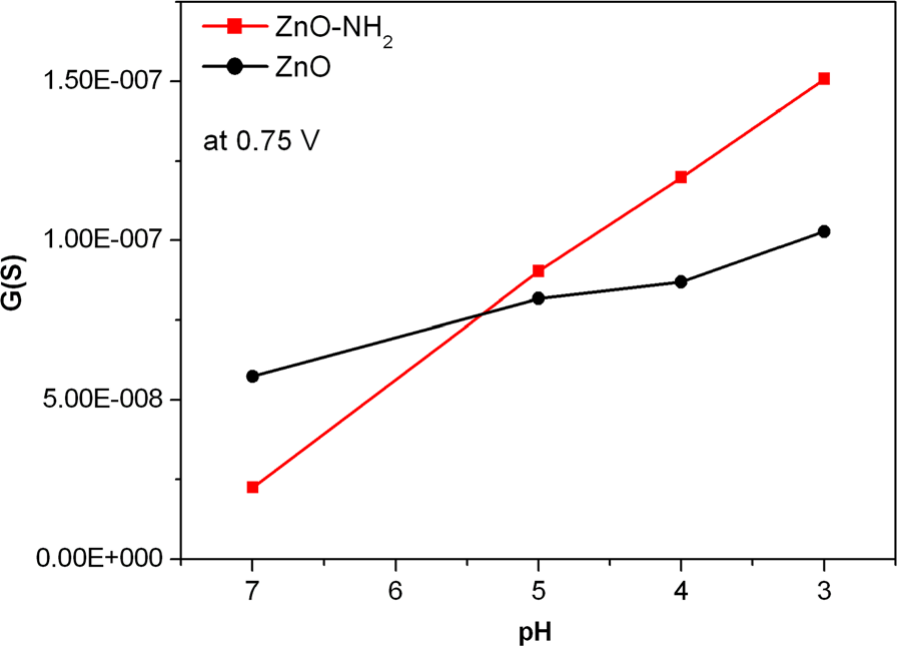
**Conductance (*G*) values at 0.75 V for the ZnO-gold junction at different pH values.** The bare ZnO wire is plotted in black, and ZnO-NH_2_ in red. The lines are a guide for the eyes.

The experimental *I*-*V* curve of the unfunctionalized ZnO-gold junction (Figure 5b) shows a tiny shift from the initial neutral condition (relative shift 85.3 nA at 2 V) which is consistent with the literature results [23]. To additionally prove the superiority in pH response of the amine-functionalized material with respect to the non-functionalized ZnO wire, the conductance *G* of both gold-ZnO junctions was calculated at 0.75 V, thus in the linear region of the *I*-*V* characteristics. The plot of the conductance values is reported as a function of the pH in Figure 6, showing that the pH dependence is almost linear for both samples in the pH range from 3 to 7. However, the conductance of the bare ZnO wire (in black) shows a reduced slope with respect to the ZnO-NH_2_ wire (in red), thus suggesting that the amine-functionalized ZnO wire could function as an effective pH sensor on the developed nanogap platform.

The pH-dependence conduction of ZnO wires is attributed to the formation of the hydroxyl groups during the acidification step, leading to a pH-dependent net surface charge, changing the voltage at the metal oxide/liquid interface [23]. Here, in the presence of amine-functionalized ZnO wires, the acidification leads to the protonation of the amine groups (from NH_2_ to NH_3_^+^, Figure 1) in addition to the ZnO surface charges. The large amount of amine groups in the functionalized sample is responsible for the stronger conductance variation of single gold-oxide-gold junction.

## Conclusions

In conclusion, we demonstrated that the amine-functionalized ZnO microwire showed a dramatic variation in conduction when exposed to acidic pH variation. The amine-functionalized microwires exhibited a stable and repeatable operation at different pH values, resulting in a remarkable sensitivity to relatively small changes in the pH values of the liquid. This system can work in liquid or dry conditions, i.e., after drying the deposited liquid drop or after immersion in a liquid system, it is thus flexible, portable, and requires a small amount of liquid to operate. Since the developed junction is sensitive to the H^+^ concentration of the liquid for low values of applied voltage (around 1 to 2 V), the power consumption of the whole measuring electronics is low. In addition, the synthesis of the ZnO wires is easy, surfactant free, and scalable, and the method for gold electrode array production is cost-effective and reliable. The nanocube electronic system makes also the final system ready-to-use for *in situ* measurements. The results show not only that properly functionalized ZnO materials are promising candidates for sensing application in liquid systems, but also that this cost-effective and customized solution can be easily engineered and integrated into more complicated electronic devices.

## Competing interests

The authors declare that they have no competing interests.

## Authors’ contributions

VC carried out the synthesis, the chemical functionalization, the microwire deposition on the nanogap, all the physical-chemical characterization measurements, and drafted the manuscript. PM fabricated the nanocube, carried out the dielectrophoresis process and all the electric tests, and drafted the manuscript. DP fabricated the whole nanogap array chip by lithographic microfabrication. GP and DD participated in the design of the study and corrected the manuscript draft. VC and PM conceived, designed, and coordinated the study. All authors read and approved the final manuscript.

## Authors’ information

VC got the European PhD in Material Science and Technology in 2008 at Politecnico di Torino, Italy, and earned her masters degree in Chemical Engineering in 2004 at the same university. From 2008 to 2010, she had a post-doctoral position at the Department of Physical Chemistry, Faculty of Chemistry, University of Munich, Germany. At present, she is a researcher at the Center for Space Human Robotics of Istituto Italiano di Tecnologia in Turin, Italy. She is involved in the chemical synthesis and characterization of nanowires and nanoparticles of both polymeric and oxide-based materials for piezoelectric and sensing applications. She is an author of more than 50 peer-reviewed works in international journals. PM has a background in information technology. His expertise ranges from analog and digital electronics to embedded system design for micro and nano applications. His scientific interests are focused on nanotechnology with emphasis on nanogap production and utilization. The scope of the nanogap covers from molecular electronics, biomolecular sensing, and biomedical applications. He currently works as a programmer and a network engineer at the Department of Electronics of Politecnico di Torino, Italy. DP got in 2003 his degree in Materials Science at the Università degli Studi of Turin, Italy, and then in 2007 his Ph.D. degree in Electronic Devices at Politecnico di Torino. He joined the Center for Space Human Robotics of Istituto Italiano di Tecnologia in Turin, Italy in 2011 as a technician. He is skilful in optical lithography, wet chemical etching, and PVD techniques for thin films coatings (thermal and electron beam-assisted evaporation and sputtering). GP is a full professor from 2006 at the Department of Electronics of Politecnico di Torino (Italy) where he teaches electron devices and integrated system technology. He received his Dr. Ing. and Ph.D. degrees in Electronics Engineering in 1986 and 1990, respectively. His research activities started at the end of 1980s and were initially focused on VLSI architecture for artificial intelligence and moved during 1990s toward the physical design of VLSI systems for high-rate and high-speed transmission and coding algorithms. His current interest involves the use of nanotechnologies in integrated systems, and he is working on molecular transport for beyond CMOS structures and on molecule interaction in molecular QCA. He is also actively working on advanced microfabrication and on self-assembly techniques. He is an author of more than 100 published works. DD received his Engineering degree and his Ph.D. in Electronic Engineering at Politecnico di Torino, Italy, in 1991 and 1995, respectively. He has a full position as assistant professor at Politecnico di Torino for the ‘Bio-Micro&Nano Systems’ and ‘Nanoelectronics’ classes, and he is leading the MiNES Group (Micro&Nano Electronic Systems) at the Department of Electronics and Telecommunications (DET) of Politecnico di Torino. DD is also currently coordinating the microelectronic research line in the Center for Space Human Robotics of Istituto Italiano di Tecnologia in Turin. He is an author and a coauthor of two patents and of more than 100 scientific publications in journals and conference proceedings related to micro and nano systems.

## Supplementary Material

Additional file 1 **This file contains nitrogen sorption isotherm with BET surface area of the ZnO microwires, pH-switching partitioning of the ZnO and ZnO-NH**_**2**_** samples, and simulation details.** Click here for file
