# Supplementary material for: pH-triggered conduction of amine-functionalized single ZnO wire integrated on a customized nanogap electronic platform
Source: Nanoscale Res Lett. 2014 Jan 31;9(1):53. doi: 10.1186/1556-276X-9-53 (PMC3912924; doi:10.1186/1556-276X-9-53)
Supplement: Additional file 1 — This file contains nitrogen sorption isotherm with BET surface area of the ZnO microwires, pH-switching partitioning of the ZnO and ZnO-NH 2 samples, and simulation details. [file 1556-276X-9-53-S1.docx]

**Supporting information**

**pH-triggered conduction of amine-functionalized single ZnO wire integrated on a customized nanogap electronic platform**

V. Cauda, P. Motto, D. Perrone, G. Piccinini, D. Demarchi


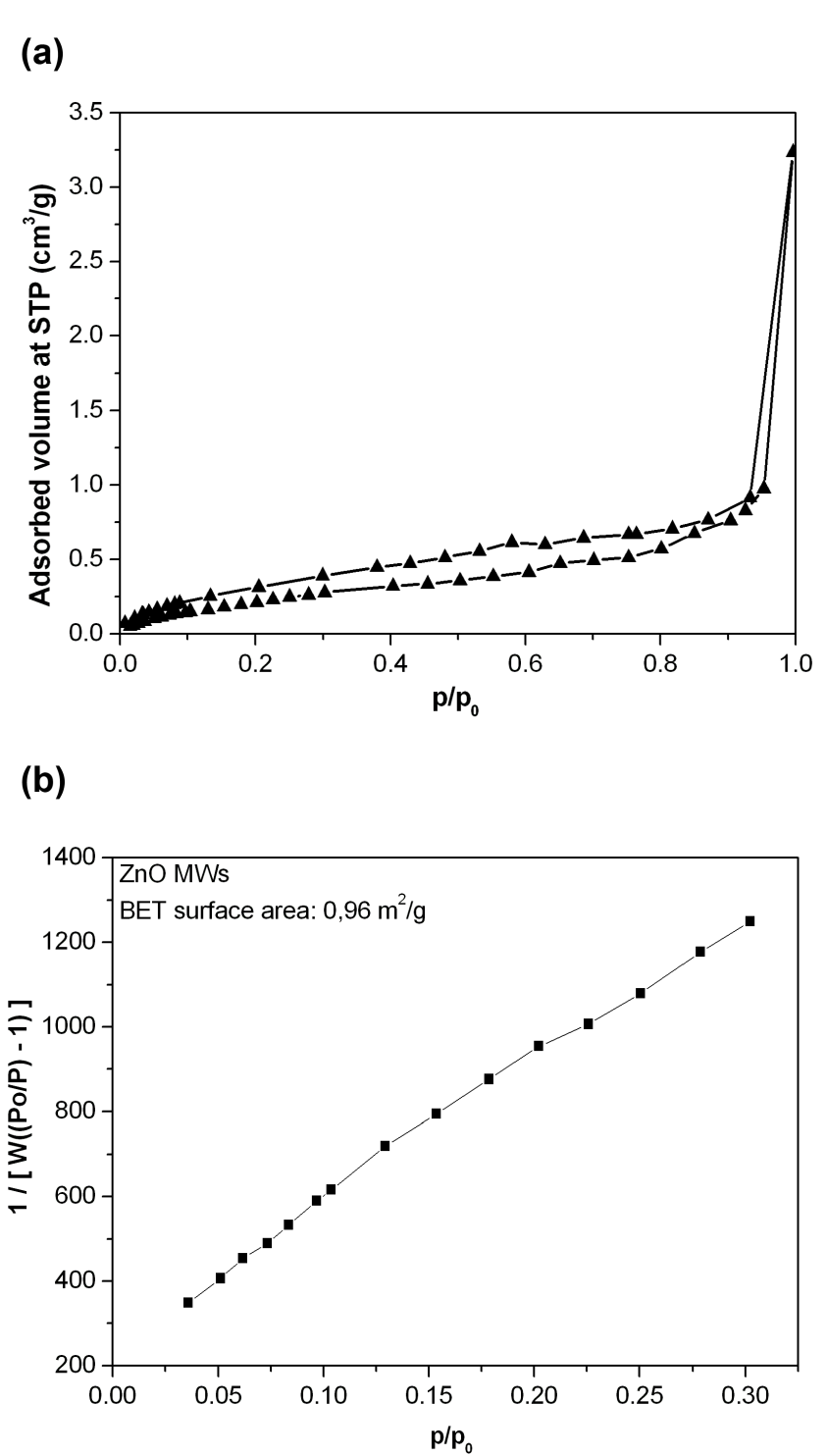


**Figure S-1.** (a) Nitrogen sorption isotherm and (b) Brunauer-Emmet-Teller (BET) plot for the estimation of the surface area related to the ZnO microwires, prior to chemical functionalization.

Nitrogen sorption isotherm (Fig. S-1a) is of type III, typical of non-porous materials. The BET surface area was measured by BET multipoint method (Fig. S-1b) within the relative pressure range of 0.1-0.3 p/p0 leading to a value of 0.96 m^2^ g^-1^.

An easy experiment to re-confirm the presence of surface charges in our three ZnO systems, relies on the pH-triggered transfer of the ZnO wires from hydrophobic to hydrophilic solutions in a biphasic system (organic solvent and water). At the beginning the two samples were immersed in water, and interestingly only the unfunctionalized ZnO wires resulted homogeneously dispersed, whereas the ZnO-NH_2_ were confined on the upper water meniscus (Fig. S-2a). The C_3_H_6_-NH_2_ chain confers to the amino-functionalized sample a hydrophobic behavior. Indeed, by adding an organic solvent (i.e. toluene) we noted a good dispersibility of the ZnO-NH_2_ material, whereas the unfunctionalized ZnO wires remained dissolved in water (Fig. S-2b). Phase-switching from the upper organic phase down to the water phase of both hydrophobic amine-functionalized ZnO wires can be triggered by adding few drops of acid (HCl 1M, Fig. S-2c). Indeed, the protonation of the amino-propyl shell on ZnO-NH_2_ wires lead to hydrophilic wires, thus driving the phase transfer at the interface between organic phase and water. Due to the micrometer size of the wires, the present results should be interpreted not as true phase transfer of the functionalized ZnO wires across toluene/water interfaces, but as pH-switched partitioning of the wires at the interface. Indeed the amine-functionalized wires are still attached at the organic/water interface during the pH variation, as visible from the absence of complete dispersion of the ZnO-NH_2_ wires in both toluene and water phases. Apart these details, these switchable states between hydrophilicity and hydrophobicity confirmed the high sensitivity of the amine-functionalized shell on ZnO wires to pH variations.


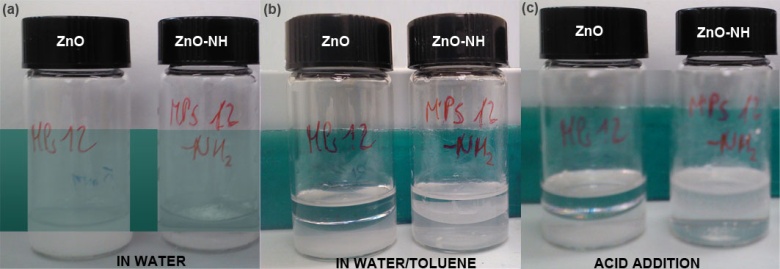


**Figure S-2.** pH-switch in water/organic biphasic system of the sample ZnO (left), ZnO-NH_2_ (right). (a) The two samples in water; (b) addition of organic phase, toluene; (c) addition of few drops of acid (HCl 1M).

**Simulation details**

The simulation procedure is described briefly as follows. First, the electronic structures of the two gold electrodes were calculated to get a self-consistent potential, which provides the real space boundary conditions for the Kohn-Sham effective potential of the central scattering region. Then from the Green’s function of the scattering region, the density matrix was obtained and hence the electron density. Thus, the DFT Hamiltonian matrix was calculated using the above boundary conditions and the electron density. This procedure was iterated until self-consistency was achieved. Moreover, the current that passes through the scattering region can be calculated from the corresponding Green’s function and self-energies using the Landauer equation:^32^

| $I\left( V_{b} \right)= \frac{2e}{h}\int_{\mu_{L}}^{\mu_{R}} dE\left( f_{L}\left( E,V_{b} \right)-f_{R}\left( E,V_{b} \right) \right)T(E,V_{b})$ | (1) |
| --- | --- |

where μ_L_ and μ_R_ are chemical potentials of the left and right electrodes respectively, f_L_(E, V_b_) and f_R_(E, V_b_) are the Fermi-Dirac functions of the two electrodes at energy E under the bias voltage V_b_, T(E, V_b_) is the bias-dependent transmission coefficient. When the bias voltage V_b_ was applied, the chemical potentials in the left/right electrode shifted and their difference is equal to eV_b_:

| $eV_{b}=\mu_{L}\left( V_{b} \right)-\mu_{R}\left( V_{b} \right)$ | (2) |
| --- | --- |

where μ_L_ = E_f_ - eV_b_/2 and μ_R_ = E_f_ + eV_b_/2, where E_f_ is the Fermi energy. For the two-probes device, the equilibrium conductance G was evaluated by the transmission coefficients T(E,V_b_) at the Fermi energy E_f_ of the system under zero bias voltage (see. Ref. 33 of the main text)

| $G=\frac{2e^{2}}{h}T(E_{f},V_{b}=0V)$ | (3) |
| --- | --- |

The wire-electrode distance was fixed as a constant for all the systems: the distance between the last plane of Au-atoms in the left electrode and the plane containing the left terminal O-atom of the wire was 1.5 Å, and the distance between the right electrode and the right terminal Zn atom plane of the nanowire was 2.3 Å. The values were obtained from total energy optimization: the axial distances of Au-O and that of Au-Zn were varied from 1 to 4 Å respectively with a minimal step of 0.1 Å. The total energies of the whole scattering region for a 3-cell system were evaluated and the minimal value was achieved at 1.5 and 2.3 Å for the left and right side, respectively.

The binding energy between the ZnO wire and Au surface was defined as:

| $E_{binding}=E\left[ nanowire+Au \right]-E\left[ nanowire \right]-E[Au]$ | (4) |
| --- | --- |

and the calculated value with the above optimized distances was -35.0 eV for the 3-cell system. Although the absolute value might be overestimated by the local density approximation (LDA) method used in our DFT electronic structure description, the negative E_binding_ denoted an exothermic process, indicating a reasonably stable and strongly coupled contact between the wires and the electrodes. The core electrons of all the atoms were modeled with Troullier-Martins nonlocal pseudopotentials, whereas the valence electrons were expanded in a double-ζ basis set. Figure 1a of the main text shows the simulated I/V plot (dotted line).
